# The possibility of a science of magic

**DOI:** 10.3389/fpsyg.2015.01576

**Published:** 2015-10-16

**Authors:** Ronald A. Rensink, Gustav Kuhn

**Affiliations:** ^1^Departments of Psychology and Computer Science, University of British ColumbiaVancouver, BC, Canada; ^2^Department of Psychology, Goldsmiths University of LondonLondon, UK

**Keywords:** magic, perception, cognition, belief updating, attention, methodology, philosophy

The past few years have seen a resurgence of interest in the scientific study of magic. Despite being only a few years old, this “new wave” has already resulted in a host of interesting studies, often using methods that are both powerful and original. These developments have largely borne out our earlier hopes (Kuhn et al., 2008) that new opportunities were available for scientific studies based on the use of magic. And it would seem that much more can still be done along these lines.

But in addition to this, we also suggested that it might be time to consider developing an outright *science of magic*—a distinct area of study concerned with the experience of wonder that results from encountering an apparently impossible event^1^. To this end, we proposed a framework as to how this might be achieved (Rensink and Kuhn, 2015). A science can be viewed as a systematic method of investigation involving three sets of issues: (i) the entities considered relevant, (ii) the kinds of questions that can be asked about them, and (iii) the kinds of answers that are legitimate (Kuhn, 1970). In the case of magic, we suggested that this could be done at three different levels, each focusing on a distinct set of issues concerned with the nature of magic itself: (i) the nature of magical experience, (ii) how individual magic tricks create this experience, and (iii) organizing knowledge of the set of known tricks in a more comprehensive way (Rensink and Kuhn, 2015). Our framework also included a base level focused on how the methods of magic could be used as tools to investigate issues in existing fields of study.

Lamont (2010) and Lamont et al. (2010) raised a number of concerns about the possibility of such a science, which we have addressed (Rensink and Kuhn, 2015). More recently, Lamont (2015) raised a new objection, arguing that although base-level work (i.e., applications of magic methods) might be useful, there is too little structure in magic tricks for them to be studied in a systematic way at the other levels, ruling out a science of magic. We argue here, however, that although this concern raises some interesting challenges for this science, it does not negate the possibility that it could exist, and could contribute to the study of the mind.

Many different kinds of magic tricks clearly exist, and Lamont (2015) provides some nice examples of these. But a science of magic centers primarily around experiential effects, not tricks (Rensink and Kuhn, 2015). The first level of our framework above the base, for instance, focuses on aspects of experience that are largely unique to magic. One such set of issues concerns the possibility of different types—and levels—of wonder; an example is the work of Griffiths (2015) on the degree of interest evoked by various magical transformations. Issues also arise around people's impression of a magical “stuff” which acts as a causal agent, and the extent to which our perceptions and beliefs can deviate from objective reality. In all of this, the details of how the experiences are evoked are irrelevant. Said another way: at this level, the scientific study of magic is not concerned with the nature of magic tricks themselves, but with the *magical aspects of experience created by these tricks*. And these aspects appear quite amenable to study.

Magic tricks are of course important, and are the focus of the next level. Here, the emphasis is on how the effects evoked in each trick (including the sense of wonder) are created. A complete trick is a complex entity, with a method that typically has multiple components. For example, a magician may use patter to set up high-level expectations, and then misdirect perception to ensure that the observer does not notice the “main” manipulations. Explorations have already begun of several such components—e.g., the manipulations underlying the French Drop (Phillips et al., 2015), the timing used in simple coin vanishes (Beth and Ekroll, 2014), the social cues in the Vanishing Ball Illusion (Kuhn and Land, 2006), and the timing needed for a Riffle Force (Olson et al., 2015). Ideally, such studies will become more powerful, knitting together our knowledge of individual components, and allowing us to understand each magic trick in its entirety.

Lamont (2015) considers magic tricks as lacking sufficient structure for this to happen. There appear to be two reasons for this concern. The first is sheer *variety*—the fact that the number of items under consideration appears “endless.” However, such variety does not of itself prevent a scientific approach to a topic. In the case of language, for instance, the number of possible sentences has exactly this “endless” character. But they can still be analyzed using approaches such as phrase-structure grammar^2^ (Chomsky, 1957) and psycho-linguistic experimentation (see O'Brien et al., 2015). In such approaches, appropriate selection of more basic elements (and their rules of combination) can let us understand aspects of a potentially infinite set of items. Methods in magic appear amenable to this, being composed of distinct components. Lamont (2015) provides a nice discussion of what some of these might be. Note that there is no problem if a component is used for different purposes in different tricks—if its analysis is based on functional considerations (as we have suggested), there will be no ambiguity in its role.

Another source of variety mentioned is a lack of clear boundaries. In this view, a trick carried out in a slightly different way is a different entity; given the nearly infinite number of small differences possible in methods (e.g., exact timing) and effects (e.g., exactly where a card appears), this results in a potentially infinite number of tricks. But this challenge has been faced—and met—in many other sciences. For example, each individual animal is different (and even changes over time). But this does not impede biology—this matter can be handled by the careful use of abstraction, with animals collected into groups of largely similar character. This approach could be readily applied to magic tricks, considering as equivalent those with little or no differences in how they are experienced—e.g., tricks in which the forcing techniques have slightly different timings, but which are equally effective.

A more interesting factor—one obliquely referred to in Lamont (2015)—is what might be called *contingency*: different methods can often achieve the same effect, and no reasons may exist as to why one method should be chosen over another. However, this might be handled by grouping together those tricks with similar effects, and focusing on the aspects common to the group. Another approach would be to *define* a particular trick as using a particular method; the issue would then reduce to one of explaining its use in a given performance. The choice made could depend on a large number of factors, such as the tricks used in the rest of the performance, or how the magician is feeling at that moment. Such contingency reflects the artistic nature of a magic performance, but does not rule out the possibility of scientific study. Given that humans respond in roughly similar ways to a given stimulus, there are stable regularities in what results *once a particular method and context have been selected*. (If this did not occur, magic could never have become a popular form of entertainment.) And such regularities can be studied in a systematic way^3^.

Regarding possibilities at the highest level of our framework (systematization), Lamont (2015) claims that the lack of structure in tricks also prevents their classification in a principled way. Note, however, that systematic analysis is just one level of our framework: even if this were somehow entirely impossible, the other levels would remain. And contrary to Lamont's assertion, we have never claimed that a science of magic requires a *complete* inventory or classification. Although, a complete inventory or classification is a laudable goal, it is not a necessary one: such systems can often be valuable even when incomplete—e.g., predicting new entities and new relationships.

But even assuming that magic tricks have little structure, would this necessarily prevent their systematic classification? Various taxonomies for magic tricks clearly exist (see e.g., Lamont and Wiseman, 1999); as such, the issue is not whether a taxonomy is possible, but how principled its organization can be. Many such systems rely on “natural kinds”—well-defined categorical entities such as chemical elements or groups of related animals (e.g., species and genera). But although natural kinds can facilitate classification, they are not necessary for this. It is entirely possible, for example, to relate in a systematic way designs described by continuous parameters, even when these parameters interact with each other in complex ways (see Woodbury, 2010).

As to how a principled classification might be created for magic tricks: this is a complex issue, involving a great amount of empirical detail. This paper (and our two earlier ones) are in some ways preliminary exercises in the philosophy of magic^4^, concerned with issues of a more general nature. But as an example of how such a venture might proceed, we have elsewhere proposed a way to classify methods of misdirection (Kuhn et al., 2014). This is based on two principles: (i) rely on psychological mechanisms as much as possible, and (ii) have the highest levels of the taxonomy center around the mechanisms affected, and not the mechanisms that control these. (For details, see Kuhn et al., 2014.) These principles greatly reduce the number of arbitrary decisions that typically enter into a classification of magic tricks (see Lamont, 2015); as such, we believe the result to be a fairly natural one. Other classifications are of course possible. For instance, some classifications may be better than ours for particular purposes, such as the teaching of prospective magicians. And even in established sciences such as biology, proposed taxonomies can vary—e.g., have more distinctions in taxonomic categories to capture more variability, or fewer distinctions to create a simpler organization (see e.g., Corliss, 1976). Finding the “sweet spot” in all of this will take time. But if history is any guide, it can be done. Our proposal—or one like it—therefore appears to have some potential to help researchers use magic to better understand perception, memory, and reasoning. And it could equally well enable knowledge of perception, memory, and reasoning to help better understand magic.

Are there factors we have not considered, factors that might influence the development of a science of magic? Undoubtedly. Will any of these ultimately prevent its development? Only time will tell. But there are grounds for optimism. For example, important advances have recently been made toward a science of film and a science of music, involving new issues that touch upon much more than just basic aspects of perception and cognition (e.g., Levitin, 2007; Ball, 2010; Shimamura, 2013; Smith, 2014). Given the nature of their subject matter, these areas are vulnerable to many of the same concerns as have been raised about a science of magic; nevertheless, the scientific development of these areas is proceeding. And if there are worries that no such attempts have ever succeeded, consider the case of steam engines. During the first century of their existence, an enormous number of these were created, with a great deal of variety and contingency in their design. And eventually, work began on a scientific framework to investigate the principles involved (see McClellan and Dorn, 2006). The resulting science—thermodynamics—has become one of the mainstays of modern physics, not only providing considerable insight into what such engines can and cannot do, but also helping us understand other processes of nature, from the metabolism of cells to the energy production of stars. Even if there is only a small chance that such a development could be possible for magic, it would appear to be a chance well worth taking.

## Conflict of interest statement

The authors declare that the research was conducted in the absence of any commercial or financial relationships that could be construed as a potential conflict of interest.

## Footnotes

^2^As discussed in Rensink and Kuhn (2015), such an area could be implemented in a variety of ways, and have various possible labels—e.g., a “science of wonder” or a “psychology of magic.” Since those issues are irrelevant to the discussion here, we will simply use “science of magic” as a general term for all of these.

^3^There is disagreement about the extent that phrase-structure grammars actually describe various languages (e.g., Postal, 1964). But this is primarily based on empirical considerations, not *a priori* ones about variety.

^4^This situation is far from unique. For example, the meaning of a word depends on its context. But this has not prevented the scientific study of language.
